# On Macula Cachectica

**Published:** 1859-09

**Authors:** James Whitehead


### EXTRACTS.

*On Macula Cachectica. By Dr. James Whitehead.* (Third Report of the Clinical Hospital, Manchester.)

*Ranking's Abstract*, 1859.

